# Targeting SPP1-orchestrated neutrophil extracellular traps-dominant pre-metastatic niche reduced HCC lung metastasis

**DOI:** 10.1186/s40164-024-00571-x

**Published:** 2024-11-11

**Authors:** Sun-Zhe Xie, Lu-Yu Yang, Ran Wei, Xiao-Tian Shen, Jun-Jie Pan, Shi-Zhe Yu, Chen Zhang, Hao Xu, Jian-Feng Xu, Xin Zheng, Hao Wang, Ying-Han Su, Hao-Ting Sun, Lu Lu, Ming Lu, Wen-Wei Zhu, Lun-Xiu Qin

**Affiliations:** 1 Hepatobiliary Surgery, Department of General Surgery, Huashan Hospital, Fudan University, 12 Urumqi Road (M), Shanghai, 200040 China; 2 Cancer Metastasis Institute, Fudan University, Shanghai, 200040 China; 3 CAS Key Laboratory of Tissue Microenvironment and Tumor, Shanghai Institute of Nutrition and Health, Chinese Academy of Sciences, Shanghai, 200031 China

**Keywords:** Pre-metastatic niche, Hepatocellular carcinoma, C-X-C motif chemokine ligand 1, Lung epithelial cells, SPP1

## Abstract

**Background:**

The mechanisms by which tumor-derived factors remodel the microenvironment of target organs to facilitate cancer metastasis, especially organ-specific metastasis, remains obscure. Our previous studies have demonstrated that SPP1 plays a key role in promoting metastasis of hepatocellular carcinoma (HCC). However, the functional roles and mechanisms of tumor-derived SPP1 in shaping the pre-metastatic niche (PMN) and promoting lung-specific metastasis are unclear.

**Methods:**

Orthotopic metastasis models, experimental metastasis models, CyTOF and flow cytometry were conducted to explore the function of SPP1 in shaping neutrophil-dominant PMN and promoting HCC lung metastasis. The main source of CXCL1 in lung tissues was investigated via fluorescence activated cell sorting and immunofluorescence staining. The expression of neutrophils and neutrophil extracellular traps (NETs) markers was detected in the lung metastatic lesions of HCC patients and mouse lung specimens. The therapeutic significance was explored via in vivo DNase I and CXCR2 inhibitor assays.

**Results:**

SPP1 promoted HCC lung colonization and metastasis by modifying pulmonary PMN in various murine models, and plasma SPP1 levels were closely associated with lung metastasis in HCC patients. Mechanistically, SPP1 binded to CD44 on lung alveolar epithelial cells to produce CXCL1, thereby attracting and forming neutrophil-abundant PMN in the lung. The recruited neutrophils were activated by SPP1 and then formed NETs-dominant PMN to trap the disseminated tumor cells and promote metastatic colonization. Moreover, early intervention of SPP1-orchestrated PMN by co-targeting the CXCL1-CXCR2 axis and NETs formation could efficiently inhibit the lung metastasis of HCC.

**Conclusions:**

Our study illustrates that HCC-lung host cell-neutrophil interactions play important roles in PMN formation and SPP1-induced HCC lung metastasis. Early intervention in SPP1-orchestrated PMN via CXCR2 inhibitor and DNase I is a potential therapeutic strategy to combat HCC lung metastasis.

**Supplementary Information:**

The online version contains supplementary material available at 10.1186/s40164-024-00571-x.

## Background

Hepatocellular carcinoma (HCC) accounts for approximately 75–85% of primary liver cancers and is the third leading cause of cancer death worldwide and the second leading cause of cancer death in China [1, 2]. Many patients lose opportunities for curative therapies due to extrahepatic metastasis, which makes the prevention of metastasis a critical component of systemic HCC treatment and the identification of pivotal factors in HCC metastasis is highly important for prolonging patient survival [3]. Thus, it is imperative to promptly identify patients at high risk of metastasis and intervene before the onset of detectable metastasis [4].

As an early event of metastasis, the pre-metastatic niche (PMN) plays a critical role in organic-specific cancer metastasis, and targeting the PMN is an effective anti-metastatic strategy for many cancers [5, 6]. The process of PMN formation involves three components: primary tumor-derived factors or extracellular vesicles (EVs), tumor-mobilized bone-marrow-derived cells and organ-specific host cells [7]. Interestingly, the diverse components and characteristics of the PMN in different organs may help explain the organ-specific metastasis [6, 8]. Notably, the interaction between tumor-secreted factors and diverse host cells plays a pivotal role in the development of metastatic organotropism in numerous cancer types [9]. However, the mechanism of PMN formation in the lung metastasis of HCC is less clear.

SPP1 is a secreted protein that is overexpressed in various solid malignancies. In HCC, SPP1 has been identified as a leading pro-metastasis gene [10]. The plasma SPP1 level shows a significant association with the number of tumors, Edmondson’s grade, serum AFP level, and especially the TNM stage. Meanwhile, elevated plasma SPP1 level was an independent prognostic factor for overall survival (OS) and disease-free survival (DFS) [11, 12]. SPP1 provides an immunosuppressive environment to support HCC growth by influencing macrophage infiltration and polarization in the primary lesion [13]. Moreover, SPP1 is recognized as a pivotal factor influencing the tumor microenvironment landscape [14]. In breast cancer, tumor-secreted SPP1 induces fibroblasts to differentiate into myofibroblasts, which subsequently facilitate epithelial-mesenchymal transition (EMT) and the metastasis of tumor cells through the secretion of CXCL12 [15]. Furthermore, in glioblastoma and esophageal squamous cell carcinoma, SPP1 promotes the infiltration of M2 macrophages and is strongly correlated with poor patient prognosis [16, 17]. However, cancer metastasis is a multistep cascade, and the function of SPP1 in distant organs and whether SPP1 regulates the PMN to fuel organ-specific metastasis of HCC is still unclear. Moreover, due to the multiple physiological functions of SPP1, directly targeting SPP1 to prevent HCC metastasis is still a major challenge. Therefore, clarifying the role of SPP1 in the metastatic process, especially in the PMN stage, may provide a potential therapeutic strategy.

Neutrophils are the fastest mobilized immune cells and constitute the only type of immune cell that independently predicts poor prognosis in patients with HCC [18, 19]. Neutrophils play dual roles in promoting or combating tumor progression through immunosuppression, reactive oxygen species, angiogenesis, or nitric oxide release in different stages of cancer metastasis [20–25]. As a product of the distinctive cell death process of neutrophils, neutrophil extracellular traps (NETs) contain granule proteins, DNA, and histones and are widely recognized because of their essential role in tumor metastasis. We previously reported that under inflammatory conditions, NETs could trap HCC cells and trigger their metastatic potential by inducing their resistance to cell death and increasing their invasiveness [26]. However, the relationship between primary tumors and NETs formation in the lung, the most common extrahepatic HCC metastasis organ, is poorly understood.

In this study, we demonstrated the crucial role of the cascade reaction among tumor-derived SPP1, lung epithelial cells and neutrophils in the formation of the PMN and HCC lung metastasis. Specifically, tumor-derived SPP1 acted on lung epithelial cells via the CD44/CXCL1 axis to increase local neutrophil infiltration. Recruited neutrophils formed abundant NETs to trap HCC cells and increase their metastatic capacity. Moreover, we found that SPP1 was correlated with MPO-DNA levels in HCC patients, which provided theoretical support for the prediction of HCC lung metastasis by measuring plasma SPP1 levels and expanding the range of plasma SPP1 applications. Moreover, early intervention in SPP1-orchestrated PMN by targeting the CXCL1-CXCR2 axis and NETs is a promising strategy to prevent HCC lung metastasis.

## Methods

### Human specimens, animal models, and cell lines

In this study, we collected four cohorts, and all patients were diagnosed as HCC according to histological examination. The tissues and plamsa samples of HCC patients were obtained from Hepatobiliary Surgery, Huashan Hospital, Fudan University with informed patient consent in accordance with a protocol approved by the Ethics Committee of Huashan Hospital, Fudan University (Shanghai, China). The first cohort included non-lung metastatic HCCs (NLM, n = 18) and lung metastatic HCCs (LM, n = 9), and the lung metastasis occurrence was diagnosed by a typical imaging appearance in chest CT scans. Another independent cohort of plasma samples (n = 21), used for myeloperoxidase (MPO) and SPP1 concentration correlation analysis, were obtained from HCC patients without radiotherapy, chemotherapy, or curative hepatectomy. Another independent cohort of lung metastatic HCC samples (n = 12) was obtained from patients who underwent metastasectomy for pulmonary metastatic lesions. The tissue microarray and cohort (n = 90) used for RNAscope In Situ Hybridization and OS/RFS analysis were purchased from Shanghai Outdo Biotech Company (Cat# HLivH180Su14, Shanghai, China). For animal experiments, 6–8 weeks old of male C57BL/6, BALB/c and BALB/c nude mice were purchased from GemPharmatech Co., Ltd (Jiangsu, China) and fed in a pathogen-free vivarium at 25° C temperature, 50–60% moisture. All animal experiments were performed following the guidelines for the care and were approved by the institutional review board of the Department of Laboratory Animal Science, Fudan University (Shanghai, China). The mouse alveolar epithelial cell line MLE-12 was purchased from Procell Life Science & Technology (Wuhan, China); the HEK293T cell line, mouse HCC cell line H22, and human non-small cell lung cancer cell line A549 were obtained from the Chinese Academy of Science (Shanghai, China). Mouse HCC cell line Hepa1-6 and human HCC cell line MHCC97H were obtained from the Liver Cancer Institute, Fudan University (Shanghai, China). HEK293T, Hepa1-6, MHCC97H and A549 were cultured in DMEM (Gibco, Cat# 11965118) supplemented with 10% FBS (Gibco, Cat# 10270106) and 1% penicillin and streptomycin (Beyotime, Cat# C0222) at 37 °C in 5% CO_2_. MLE-12 was cultured in DMEM/F12 (Gibco, Cat# 11320033) supplemented with 2% FBS and 1% penicillin and streptomycin at 37 °C in 5% CO_2_.

### Mice model: SPP1-modified metastasis and treatment model

Hepa1-6^vector/SPP1^ CM (500 μl/mouse, QD, intraperitoneally (i.p)) or SPP1 protein (10 μg/mouse, QD, i.p) was injected for 7 days before tumor cell implantation to imitate a cancer-related inflammation condition prone to generate NETs. A neutrophil-depleting antibody (12.5 μg/mouse, QD, i.p, Biox Cell, Cat# BE0075-1) was given at the same time as CM or SPP1 to eliminate neutrophils. Anti-SPP1 neutralizing antibody (2 μg/mouse, QD, i.p, R&D System, Cat# AF808) was given at the same time as CM or SPP1 to neutralize secretory SPP1. For the treatment exploration, DNase I (200 U/mouse, QD, i.p, Solarbio, Cat# D8071), GSK484 (5 mg/kg, twice-weekly, i.p, MCE, Cat# HY-100514), and SB225002 (2 mg/kg, QD, i.p, MCE, Cat# HY-16711) were given to degrade NETs and inhibit neutrophil recruitment, separately.

### Mice model: establishment of spontaneous metastasis in SPP1-modified models

In 7 days after establishment of the SPP1-modified model in mice, the subcutaneous xenograft tumors were removed and minced into 1–2 mm^3^ sections and washed in precooled 1✕PBS. Then, mice were anesthetized with intraperitoneal injection of tribromoethanol (250 mg/Kg, Selleck, Cat#S4508) and laparotomy with a 3–4 cm incision along the xiphoid process was made to expose the left hepatic lobe. Using a sterile cotton swab to gently roll out the left hepatic lobe and keeping it moist with normal saline. The minced tumor fragment was directly inserted into the subcapsular parenchyma of the left hepatic lobe using sterile ophthalmic tweezer, followed by closure of the abdominal incision with layered sutures. Mice were sacrificed after 5 weeks and the tumors, normal liver and lung were removed. Specimens were fixed in 4% paraformaldehyde (PFA, Beyotime, Cat# P0099), embedded into paraffin blocks, or flash-frozen in liquid nitrogen and stored at − 80 °C to obtain tissue protein or RNA. Serial sectioning of the lung was performed, and H&E staining was used for the determination of metastatic burden.

### Mice model: establishment of experimental metastasis in SPP1-modified models

In 7 days after the establishment of the SPP1-modified model in mice, 2 × 10^6^ Hepa1-6 cells were intravenously (i.v) injected into C57BL/6, 2 × 10^6^ MHCC97H cells were i.v injected into BALB/C nude mice and 1 × 10^6^ H22 cells were i.v injected into BALB/C mice to form experimental metastasis model, and the in vivo fluorescence imaging was performed using In vivo imaging system (IVIS) Spectrum (PerkinElmer) with Living Image software. The mice were sacrificed and lung metastasis burden was assessed in day 35, day 40 and day 16, separately. The percentage of metastasis events or the number of lung metastatic lesions was directly assessed on lung sections using H&E staining.

### CyTOF

CyTOF analysis was conducted by PLT-Tech Inc. (Hangzhou, China), involving the dissociation of lung tissue into single cells using DNase I, collagenase IV, and hyaluronidase. The cells were then stained with Pt-194 and incubated with DNA Intercalator-Ir to distinguish between singly nucleated cells and doublets. Subsequently, a permeabilization buffer was utilized, and the cells were subjected to an intracellular antibody panel before being rinsed. Subsequently, the cells underwent rinsing, and the signals were analyzed utilizing a CyTOF system (Helios™, Fluidigm). Identification of immune cell types was achieved through nonlinear dimensionality reduction (t-distributed stochastic neighbor embedding), followed by density clustering. The antibodies for CyTOF were listed in Table S1.

### Neutrophil isolation and NETs formation

The procedure of neutrophil isolation and NETs formation was described in our previous study [26]. Briefly, to isolate mouse neutrophils, bone marrow cells were harvested in sterile Hank’s balanced salt solution (Beyotime, Cat# C0218) and gently laid on the top of a 2-layer Percoll gradient (65% and 55%, Cytiva, Cat# 17089101), followed by centrifugation at 1000 *g* for 30 min at room temperature (RT) without brake. Neutrophils were collected from the interface of 65% and 55% fractions. To isolate human neutrophils, whole blood samples were freshly collected from HCC male volunteers with heparinized tubes and laid on top of Polymorphprep (Axis Shield, Cat# 1114683) and centrifuged at 500 *g* for 30 min at RT without brake. For NETs formation, freshly isolated neutrophils were seeded on poly-L-lysine (Beyotime, Cat# C0313) coated coverslips (NEST, Cat# 801007) in 24-well plates and stimulated with recombined human or mouse SPP1 for 4–6 h to form NETs. In NETs formation under CM stimulation assays, a 1:1 mixture of RPMI 1640 (Gibco, Cat# 61870036) and CM was added. In all assays, 20 nM PMA (Sigma, Cat# P8139) was used as a positive control while 1XPBS was a negative control. Next, coverslips were stained with SytoxGreen (Invitrogen, Cat# S7020) and Hoechst (Beyotime, Cat# C1029) for 20 min. Zeiss inverted fluorescence microscopes (Leica) were used to detect and image fluorescence. In some situations, NETs were fixed with 4% PFA and stained with DAPI (Beyotime, Cat# C1002) and SytoxGreen for further IF detection.

### Migration assays

The procedure of two-chamber migration assays was previously described [21]. For neutrophil migration assay, 5 × 10^5^ freshly isolated neutrophils in serum-free RPMI 1640 were added to the upper chamber (3.0 µm, Corning, Cat# 3415) and a 1:1 mixture of RPMI 1640 and CM was added to the lower chamber as the chemoattractant and incubated at 37 °C in 5% CO_2_ for 3 h. As for tumor cell migration assay, 4 × 10^4^ Hepa1-6 cells in serum-free DMEM were added to the upper chamber (8.0 µm, Corning, Cat# 3422) and DEME (supplemented with 10% FBS) was added to the lower chamber and incubated at 37 °C in 5% CO2 for 48 h. The migrated cells in the lower chamber were fixed by 4% PFA fix solution (Beyotime, Cat# P0099) and then counted by 0.2% crystal violet staining (Beyotime, Cat# C0121).

### Immunofluorescence staining (IF)

The day before staining, cells were seeded on coverslips in 24-well plates and treated with indicated conditions. Subsequently, the coverslip was washed by 1XPBS for 3 times and fixed with 4% PFA for 20 min at RT. Then coverslips were permeabilized with 0.1% Triton X-100 (Sigma, Cat# X100) for 15 min at RT, washed with 1✕PBS, and blocked with PBS containing 1% BSA (Beyotime, Cat# ST023) for 1 h at RT. Staining the coverslips with the primary antibody in blocking buffer at 4 °C overnight, the antibodies were listed in Table. S4. After washing, coverslips were incubated with corresponding fluorescence-conjugated secondary antibodies for 1 h protected from light. DAPI was used for followed nuclear staining. Zeiss inverted fluorescence microscopes (Leica) were used to detect and image fluorescence and images were analyzed with ImageJ software.

### Quantification and statistical analysis

Datasets were analyzed with GraphPad Prism software. The student’s t-test, Chi-squared test, Fisher exact test, and one- or two-way ANOVA test were used to analyze the data as in the figure legends. Differences were considered significant at  *P*< *0.05*.

## Results

### Tumor-secreted SPP1-orchestrated PMN favors HCC lung metastasis

**Fig. 1:**
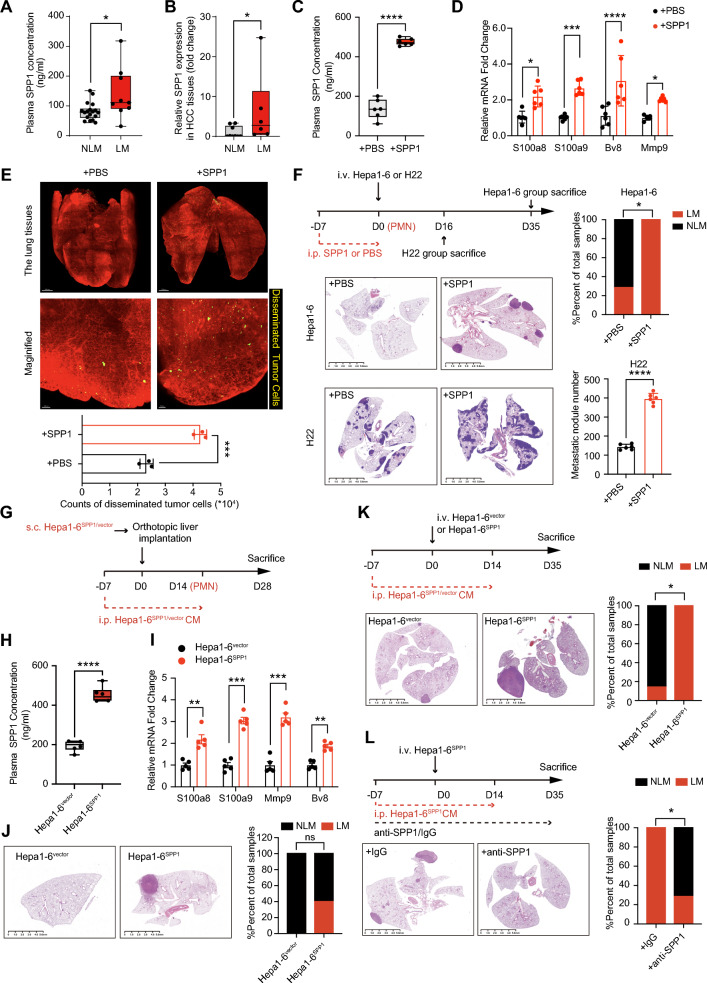
SPP1 is closely associated with the lung metastasis of HCC. **A** ELISA detection of plasma SPP1 concentration in samples of NLM (n = 18) and LM (n = 9). **B** qRT‒PCR analysis of SPP1 mRNA levels in tissues of NLM (n = 9) and LM (n = 6). The *P* values were calculated by Student’s *t*-test. **C**, **D** SPP1 protein levels in plasma (**C**) and pro-metastatic gene expression in the lungs (**D**) of mice at 7 days after SPP1 treatment (n = 6 per group)**.** The *P* values were calculated by Student’s *t*-test. **E** Tissue-clearing technique showed early colonization of Hepa1-6^GFP^ in the lungs at 1 day after i.v. injected in PBS or SPP1 pre-treated mice (n = 3 per group). The *P* values were calculated by Student’s *t*-test. **F** The effect of SPP1 on HCC metastasis was evaluated in the SPP1-PMN model of Hepa1-6 (n = 7 per group) and H22 cell lines (n = 6 per group). The *P* values were calculated by two-tailed Fisher exact test (for Hepa1-6) and Student’s *t*-test (for H22), respectively. **G**–**J** Experimental schematics of modified orthotopic spontaneous metastasis model (**G**). Quantification analysis of SPP1 protein levels in plasma (**H**) and pro-metastatic gene expression in the lungs (**I**) of mice at 14 days after orthotopic liver transplantation (n = 5 per group). Shown were representative images of lung metastatic loci of Hepa1-6^vector/SPP1^ in the SPP1-orchestrated spontaneous metastasis model (**J**, n = 5 per group). The *P* values were calculated by a student’s *t*-test (for H-I) and two-tailed Fisher exact test (for J), respectively. **K** I.v. injection of Hepa1-6^vector^ or Hepa1-6^SPP1^ cells with matched CM-treatment for lung metastasis analysis. Representative images and the ratio of lung metastasis were shown (n = 7 per group). The *P* values were calculated by the two-tailed Fisher exact test. **L** The effect of anti-SPP1 neutralizing antibody on HCC metastasis was evaluated in the experimental metastasis model (n = 7 per group). The *P* values were calculated by the two-tailed Fisher exact test. For all tests, significance is determined with a 95% confidence interval (ns, P > 0.05; *P < 0.05; **P < 0.01; ***P < 0.001; ****P < 0.0001)

To assess the potential role of SPP1 in the lung metastasis of HCC, we initially validated the upregulation of SPP1 mRNA expression in the TCGA-HCC cohort (Figure S1A-B). A significant increase in SPP1 expression in liver tumor tissues compared with that in corresponding noncancer tissues was confirmed by quantitative real-time polymerase chain reaction (qRT-PCR) (Figure S1C). Considering that SPP1 can be secreted by various matrix cells in the tumor microenvironment, we detected SPP1 expression via RNAscope In Situ Hybridization and demonstrated that the tumorous secretion of SPP1 was dominant (Figure S1D-F). Moreover, SPP1 expression was positively associated with reduced OS and RFS in HCC patients according to the results of RNAscope In Situ Hybridization (Figure S1G). To further elucidate the relationship between SPP1 and HCC metastasis, we analyzed a Huashan cohort of HCC patients with lung metastasis (LM) or non-lung metastasis (NLM) (Table S2). Interestingly, SPP1 expression in HCC tissues was elevated in the LM group compared with the NLM group, and the LM group presented higher SPP1 concentrations in the circulation (Fig. 1A, B). Meanwhile, higher SPP1 expression levels in HCC tissues were positively correlated with plasma SPP1 concentration (Figure S1H). In the multivariate analysis, SPP1 was identified as an independent risk factor for lung metastasis (Table S3). Together, these results indicate that SPP1 upregulation is closely associated with lung metastasis in HCC.

Given the important role of the PMN in the metastatic colonization of tumor cells [8], we further investigated the potential effects of tumor-derived SPP1 on PMN formation and HCC lung metastasis. We initially procured recombinant mouse SPP1 (Figure S1I) and administered it to immunocompetent mice. Circulating levels of SPP1 in the murine models reached a steady state 7 days after the intraperitoneal injection (i.p.) of SPP1, similar to what has been observed in clinical pathological conditions (Fig. 1C) [12]. The RNA sequencing (RNA-seq) and qRT-PCR data revealed increased expression of various PMN-related genes and inflammation-associated factors in pulmonary tissue 7 days after i.p. injection of SPP1 (Fig. 1D, Figure S1J, Table S4). Notably, these alterations were not detected in other organs (Figure S1K-M), indicating a profound pro-metastatic remodeling effect of SPP1 on the lung (referred to as SPP1-PMN). PMN fosters early tumor cell colonization, a process that is instrumental in facilitating metastasis. Similarly, SPP1-PMN was observed to enhance early colonization of murine Hepa1-6^GFP^ cells by intravenous injection (i.v) (Fig. 1E) [27], which was concomitant with an increase in experimental metastasis in both i.v. injected Hepa1-6 and H22 cells, a suspended murine HCC cell line from malignant ascites (Fig. 1F). To better mimic the continuous and dynamic nature of PMN remodeling by tumorous SPP1 during HCC progression and the subsequent metastasis cascade, we overexpressed SPP1 in Hepa1-6 cells (Figure S1N-O) and established a modified Hepa1-6^SPP1^ or Hepa1-6^vector^ orthotopic spontaneous metastasis model with matched conditioned medium (CM) to accelerate lung remodeling [28]. We harvested lung tissues from this model 2 weeks after Hepa1-6 implantation (Fig. 1G), a timepoint generally recognized as the PMN stage (referred to as Hepa1-6^SPP1^- or Hepa1-6^vector^-PMN) [29, 30]. PMN was readily remodeled by SPP1 overexpression, as evidenced by the higher circulating SPP1 level and PMN-related genes in Hepa1-6^SPP1^-PMN than in the control group (Fig. 1H, I), mirroring the scenario observed with SPP1 administration. Consistent with the remodeling of the PMN, SPP1 overexpression was also accompanied by a greater frequency of spontaneous pulmonary metastasis (Fig. 1J), as opposed to metastasis in other organs. Moreover, overexpression of SPP1 in Hepa1-6 had no effect on the subcutaneous and orthotopic tumor growth (Figure S1P). Notably, similar to SPP1-PMN, Hepa1-6^SPP1^ CM alone also efficiently led to an increased experimental metastasis through PMN remodeling (Fig. 1K). To further prove that the pro-metastatic effects of SPP1 were mediated through PMN remodeling rather than augmenting the invasiveness of the primary tumor, we generated an N-terminal signal peptide deleted SPP1 mutant in Hepa1-6 (Hepa1-6^SPP1△1–48^, also known as SPP1 isoform-2), which preserves the intracellular expression and bioactivity of full-length SPP1 while simultaneously inhibiting its extracellular secretion (Figure S2A-B) [31]. In vitro, both the overexpression of SPP1 and SPP1△1–48 had a negligible effect on the proliferation and migration of Hepa1-6 cells (Figure S2C-E), which minimized the possible autocrine or paracrine effects of SPP1 on HCC tumor cells directly. Nonetheless, the attenuation of SPP1 secretion with SPP1△1–48 significantly reduced the lung metastasis as compared with the full-length SPP1 isoform but had minimal influence on the growth of subcutaneous and orthotopic tumors (Figure S2F-G). Moreover, systemically neutralizing SPP1 effectively mitigated the remodeling of PMN remodeled by Hepa1-6^SPP1^ CM and attenuated the augmented experimental metastasis of i.v Hepa1-6^SPP1^ cells (Fig. 1L). This effect was also validated in an immunodeficient mouse model, where SPP1 neutralization reduced experimental lung metastasis induced by MHCC97H, a highly metastatic human HCC cell line with robust high SPP1 expression (Figure S2H). Taken together, these results indicate that secretory SPP1 plays a dominant role in the lung metastasis of HCC accompanied by PMN remodeling.

### Neutrophils are essential in SPP1-orchestrated lung PMN

**Fig. 2:**
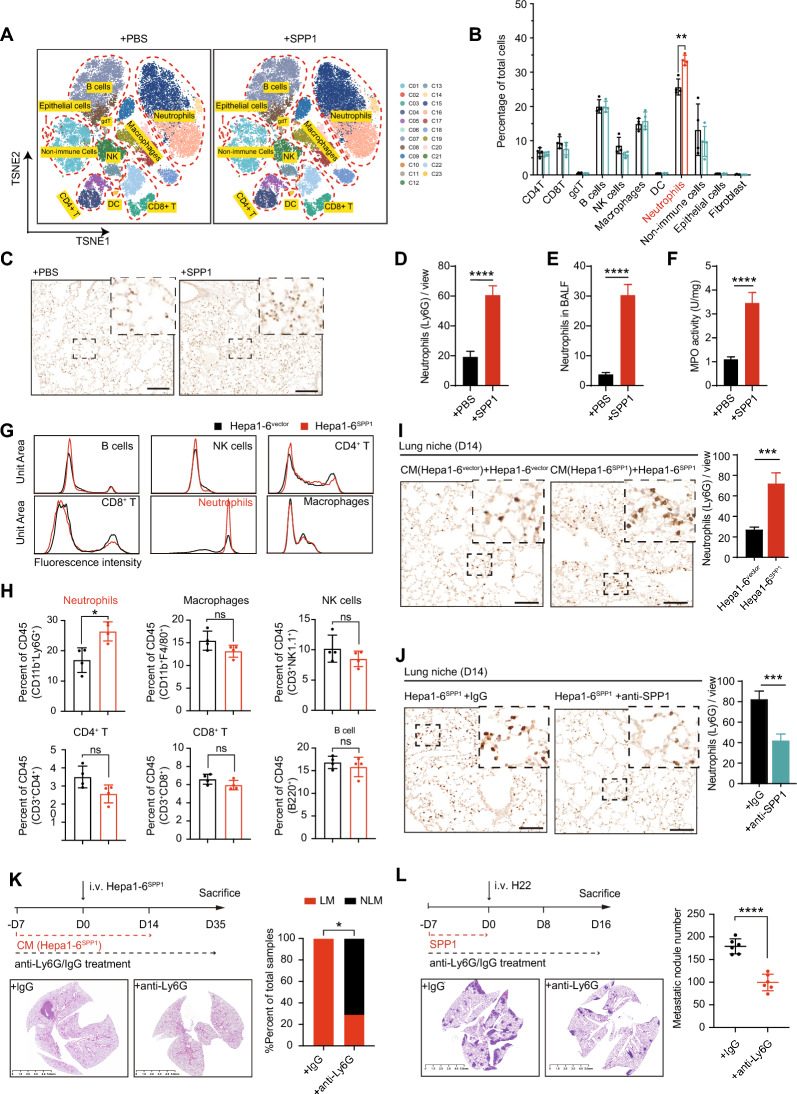
SPP1 promotes HCC lung metastasis in a neutrophil-dependent manner. **A** TSNE plot projection of single cells from PBS-PMN and SPP1-PMN, colored by 23 clusters. The lungs were harvested after i.p. injecting SPP1 for 7 days (related to Fig. 1D) (n = 4 per group). **B** The quantification of the distinct immune cell subtypes in PBS- and SPP1-PMN group. **C**, **D** IHC staining of neutrophils (Ly6G^+^) in the lung tissues (**C**). To verify, the number of neutrophils is counted in five random fields and took up the average per sample (**D**, n = 5 per group). The *P* values were calculated by a student’s *t*-test. Scale bar, 100 μm. **E**, **F** FC analysis of neutrophils (CD45^+^CD11b^+^Ly6G^+^) in the BALF and MPO activity in the lungs from SPP1-PMN model (n = 5 per group). The *P* values were calculated by a student’s *t*-test. **G**, **H** FC analysis of immune cells in lung tissues from the orthotopic model of Hepa1-6^vector/SPP1^ mice (n = 4 per group). Lungs were harvested at 14 days after orthotopic inoculation. Percentages of neutrophils (CD45^+^CD11b^+^Ly6G^+^), macrophages (CD45^+^CD11b^+^F4/80^+^), NK cells (CD45^+^ NK1.1^+^), CD4 + T cells (CD45^+^CD3^+^CD4^+^CD8^−^), CD8 + T cells (CD45^+^CD3^+^CD4^−^CD8^+^), B cells (CD45^+^B220^+^) were shown. The *P* values were calculated by a student’s *t*-test. **I**, **J** Representative IHC images and quantification of Ly6G staining and in the lungs of Hepa1-6 orthotopic implantation model and anti-SPP1 treatment model (n = 5 per group). Scale bar, 100 μm. The *P* values were calculated by a student’s *t*-test. **K**, **L** Representative H&E images and quantification of lung metastasis loci of Hepa1-6^SPP1^ (**K**) or H22 (**L**) in SPP1-associated experimental metastasis model after depleting neutrophils by anti-Ly6G antibody. For all tests, significance is determined with a 95% confidence interval (ns, P > 0.05; *P < 0.05; **P < 0.01; ***P < 0.001; ****P < 0.0001)

To further elucidate the mechanism by which SPP1 orchestrated lung PMN, we initially conducted mass cytometry (CyTOF) with 42-metal isotope-tagged monoclonal antibody to evaluate the alterations in all major immune cells and certain stromal cell types in the lung after SPP1 administration for 7 days (SPP1-PMN, related to Fig. 1D). We identified 23 distinct cellular clusters and the following principal cell subsets based on the expression levels of lineage markers: CD8^+^ T cells, CD4^+^ T cells, B cells, macrophages, gamma-delta T cells, natural killer cells, neutrophils, dendritic cells, fibroblasts, and epithelial cells (Fig. 2A, Figure S3A-B). Compared with the control group, the SPP1-PMN group presented a significant increase in the neutrophil population, whereas no discernible changes were observed in other immune cell clusters (Fig. 2B). In accordance with the CyTOF results, flow cytometry (FC) revealed that neutrophils were the dominant cell type whose abundance was altered in SPP1-PMN (Figure S3C-D). Moreover, the ImmuCC algorithm applied to RNA-seq data of SPP1-PMN (related to Figure S1J) also predicted a pronounced trend toward increased neutrophils, but not other cells (Figure S4A) [32, 33]. Immunohistochemistry (IHC) analysis of pulmonary neutrophils (Fig. 2C, D), FC analysis of bronchoalveolar lavage fluid (BALF) (Fig. 2E), and myeloperoxidase (MPO) activity analysis (Fig. 2F) further confirmed that the number of neutrophils was strongly increased in SPP1-PMN.

In addition to the setting of SPP1-PMN, FC analysis of Hepa1-6^SPP1^-PMN confirmed a considerable increase in the neutrophil population, alongside a varied reduction in other immune cells including macrophages, natural killer cells, T cells, and B cells, compared with that of Hepa1-6^vector^-PMN (Fig. 2G, H), and IHC confirmed the increase in neutrophils in the Hepa1-6^SPP1^ group (Fig. 2I). Moreover, a marked increase in the number of pulmonary neutrophils was observed in Hepa1-6^SPP1^ CM-treated lungs, and this increase was dampened by the SPP1 neutralizing antibody (Fig. 2J), which was consistent with the change in metastasis (Fig. 1L). To further determine the necessity of neutrophils in SPP1-orchestrated PMN, we used an anti-Ly6G antibody to deplete neutrophils within lung PMN induced by Hepa1-6^SPP1^ CM or SPP1 and observed a significantly mitigated experimental lung metastasis of Hepa1-6 and H22 cells, respectively (Fig. 2K, L). Similarly, the experimental metastasis of human MHCC97H cells was also abrogated by neutrophil depletion (Figure S4B). Taken together, these findings strongly indicate that neutrophils are essential in SPP1-orchestrated lung PMN.

### Neutrophil recruitment to SPP1-orchestrated PMN depends on lung epithelial CXCL1 secretion

**Fig. 3:**
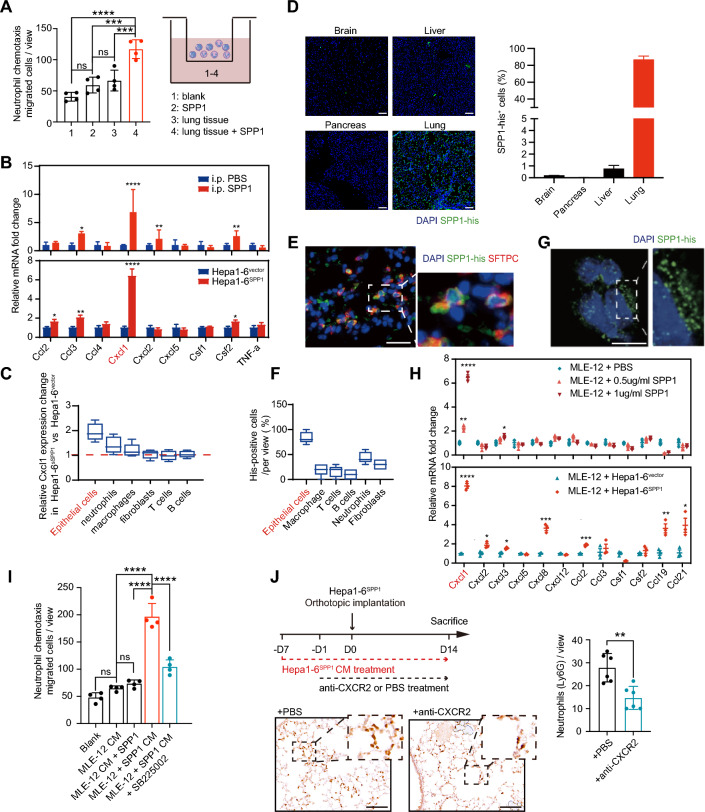
SPP1 promotes neutrophil recruitment by stimulating lung epithelial cells to increase CXCL1 secretion. **A** CM from different groups was placed in the lower chambers. Primary neutrophils were isolated and then placed in the upper chambers and their migration into the lower chambers was counted. The *P* values were calculated by one-way ANOVA. **B** qRT-PCR showed the expression of neutrophil-related chemokines and cytokines in lung tissue in SPP1-PMN (upper panel, related to Fig. 1C) and in Hepa1-6^vector/SPP1^-PMN (lower panel). The *P* values were calculated by Student’s *t*-test. **C** qRT‒PCR analysis of *Cxcl1* expression in epithelial cells, neutrophils, macrophages, fibroblasts, T cells, and B cells purified from the lung tissues in the Hepa1-6^vector/SPP1^-PMN model. **D** Representative images (right panel) and quantitative analysis of SPP1-his distribution in different tissues of mice with i.p. administration of SPP1-his. Scale bar, 50 μm. **E**, **F** IF staining of SPP1-his and lung epithelial cell (SFTPC^+^) colocalization (E) and SPP1-positive rates (F) in different cells of mice with i.p. administration of SPP1-his (related to Figure S5C, Supporting Information). Scale bar, 20 μm. **G** Representative IF image of SPP1-his internalization by MLE-12 cells. Scale bar, 10 μm. **H** qRT‒PCR showed the neutrophil-related chemokines and cytokines in MLE-12 cells treated with various concentrations of SPP1 (upper panel) or cocultured with Hepa1-6^vector/SPP1^ (lower panel). The *P* values were calculated by Student’s *t*-test. **I** Migrated cell numbers of murine neutrophils recruited by CM of MLE-12 cells, SPP1-pretreated MLE-12 cells or MLE-12 cells treated with 1 µg/ml SPP1 plus CXCR2 inhibitor (SB225002, 40 nM) or by MLE-12 CM plus 1 µg/ml SPP1. The *P* values were calculated by one-way ANOVA. **J** IHC images and quantification of neutrophils in the Hepa1-6-PMN models following CXCR2 inhibitor treatment (n = 6 per group). The *P* values were calculated by Student’s *t*-test. Scale bar, 100 μm. For all tests, significance was determined with a 95% confidence interval (ns, P > 0.05; *P < 0.05; **P < 0.01; ***P < 0.001; ****P < 0.0001)

Orthotopic implantation of Hepa1-6^SPP1^ did not significantly affect circulating neutrophil counts compared to control group(Figure S5A), whereas in vitro assays revealed that CM from SPP1-stimulated lung tissues enhanced neutrophil chemotaxis, a response not induced in the setting of CM from untreated lung tissues or SPP1 alone (Fig. 3A), which implied that SPP1-orchestrated PMN may indirectly facilitate pulmonary neutrophil recruitment, which is dependent on local lung microenvironment.

To elucidate this phenomenon, we examined the expression of several cytokines and chemokines in the Hepa1-6^SPP1^- or Hepa1-6^vector^-PMN in a modified orthotopic model, as well as the SPP1-PMN. Several neutrophil-recruiting chemokines, including C-X-C motif chemokine ligand 1 (*Cxcl1*), were significantly upregulated, whereas the expression of macrophage-related chemokines, including C–C motif chemokine ligand 1, remained unchanged (Fig. 3B, Figure S5B). We further sorted various types of resident cells in Hepa1-6^SPP1^-PMN and found that epithelial and neutrophils were the main sources of increased *Cxcl1* compared with those in the control group (Fig. 3C). To further elucidate how SPP1 recruited neutrophils through lung epithelial cells, SPP1 labeled with his-tag (SPP1-his) was purified and then i.p injected into the mice. We found that his-tagged SPP1 was predominantly distributed throughout the lung but not other organs, supporting the specific role of SPP1 in reshaping the lung PMN (Fig. 3D). Furthermore, IF staining revealed that exogenous SPP1 targeted mainly type II alveolar epithelial cells (AECII, Surfactant Protein C + cells (SFTPC^+^)), with fewer associations with neutrophils (MPO^+^ cells) and no association with other cells (Fig. 3E, F, Figure S5C), which was congruent with the results of qRT-PCR (Fig. 3C).

Upon stimulation with SPP1-his, the murine lung epithelial cell line MLE-12 was directly combined with the exogenous SPP1 (Fig. 3G). Moreover, qRT-PCR revealed that *Cxcl1* expression was significantly upregulated in MLE-12 cells after SPP1 stimulation or co-culture with Hepa1-6^SPP1^ cells (Fig. 3H). Consistently, the upregulation of *Cxcl1* was also induced by SPP1 or recombinant human SPP1 (rhSPP1) in freshly purified AEC II from mouse lungs and human A549 cells, respectively (Figure S5D-E). In accordance with elevated *Cxcl1* expression, CM from MLE-12 cells pre-treated with SPP1 exhibited a greater increase in neutrophil chemotaxis than MLE-12 CM or a combination of SPP1 and MLE-12 CM. This effect was abrogated by blockage of C-X-C motif chemokine receptor 2 (CXCR2, a well-known CXCL1 receptor) via SB225002 (Fig. 3I). In the orthotopic model, the increased neutrophil infiltration in Hepa1-6^SPP1^-PMN was also attenuated by CXCR2 blockade (Fig. 3J). Collectively, these data suggest that HCC-derived SPP1 facilitates neutrophil recruitment through stimulating CXCL1 expression by lung epithelial cells, thereby forming a neutrophil-enriched PMN that favors the lung-specific metastasis of HCC.

### SPP1 facilitates neutrophil recruitment by activating the lung epithelial CD44/STAT3/CXCL1 signaling axis in PMN

**Fig. 4:**
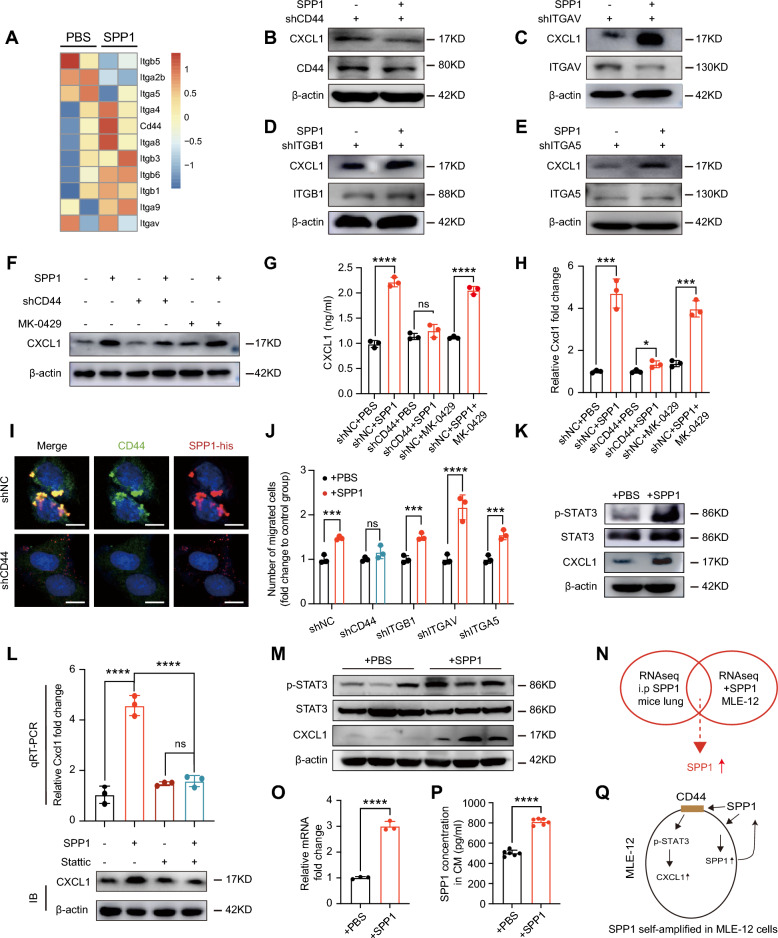
SPP1 increases CXCL1 expression in MLE-12 cells through the CD44/STAT3 axis. **A** Heatmap of the SPP1 receptor genes detected by RNA-seq in the lung tissue after i.p. administration of SPP1 for 7 days. **B**–**E** Immunoblotting of CXCL1 expression in MLE-12 shCD44 (**B**) cells, MLE-12 shITGAV (**C**) cells, MLE-12 shITGB1 (**D**) cells, MLE-12 shITGA5 (**E**) cells treated with SPP1 for 48 h. **F**–**H** Immunoblot (**F**), ELISA (**G**) and qRT-PCR (**H**) showed CXCL1 expression in MLE-12 shCD44 cells treated with SPP1 or MLE-12 cells treated with SPP1 and/or integrin inhibitor (MK-0429, 20 nM). The *P* values were calculated by a student’s *t*-test. **I** Representative IF images of SPP1-his and CD44 colocalization in MLE-12 shNC and MLE-12 shCD44 cells. Scale bar, 10 μm. **J** Relative migrated cell numbers of murine neutrophils recruited by CM of CD44-, ITGB1- or ITGAV-knockdown MLE-12 cells treated with SPP1. The *P* values were calculated by a student’s *t*-test. **K** Changes in p-STAT3, STAT3, and CXCL1 in MLE-12 cells under SPP1 stimulation. **L** qRT‒PCR (upper panel) or immune blot (lower panel) analysis of CXCL1 expression in MLE-12 cells treated with SPP1, Stattic (20 μM), and SPP1 plus Stattic. The *P* values were calculated by one-way ANOVA. **M** Immunoblotting of CXCL1, STAT3, p-STAT3 in lung tissues of mice at 7 days after PBS or SPP1 treatment. **N**–**Q** Overlapping of genes resulting from the comparison of upregulated genes in mouse lung tissue in SPP1-PMN mice model and in SPP1-pretreated MLE-12 cells (**N**). qRT-PCR (**O**) and ELISA (**P**) showed the SPP1 expression in SPP1-pretreated MLE-12 cells. The pattern diagram (Q) of MLE-12 self-amplified SPP1 expression under exogenous SPP1 stimulation. The *P* values were calculated by a student’s *t*-test. For all tests, significance was determined with a 95% confidence interval (ns, P > 0.05; *, P < 0.05; **, P < 0.01; ***, P < 0.001; ****, P < 0.0001)

SPP1 is known to interact primarily with two types of receptors, CD44 and various integrins, including αv (β1, β3, β5), α8β1, and α5β1 [34]. Our RNA-seq analysis (Figure S1J, Table S4) revealed elevated CD44 and certain integrins in SPP1-PMN, suggesting a potential role for SPP1 and its receptors (Fig. 4A) and qRT-PCR confirmed that *Cd44*, *Itgav*, *Itga5*, and *Itgb1* were the predominant SPP1 receptors in both mouse lung tissues and cultured MLE-12 cells (Figure S6A-B). Among these receptors, depletion of CD44 via lentiviral shRNA negated the SPP1-induced upregulation of CXCL1 in MLE-12 cells at both the mRNA and protein levels. In contrast, interfering with ITGB1/ITGAV/ITGA5 with neither shRNA nor the integrin inhibitor MK-0429 failed to replicate these findings (Fig. 4B–H, Figure S6C-F). IF staining confirmed the colocalization of CD44 and SPP1-his in MLE-12 cells, and the knockdown of CD44 markedly decreased SPP1 binding in vitro (Fig. 4I). Additionally, SPP1 stimulation was found to upregulate CD44 expression in MLE-12 cells (Figure S6G-H). In accordance with CXCL1 expression, the knockdown of CD44 effectively abolished the increase in neutrophil chemotaxis toward SPP1-treated MLE-12 cells, whereas the knockdown of ITGB1, ITGAV, or ITGA5 did not **(**Fig. 4J**)**, confirming that SPP1 regulated CXCL1 expression via CD44, not integrins. The Kyoto Encyclopedia of Genes and Genomes (KEGG) pathway analysis of RNA-seq data from SPP1-treated MLE-12 cells highlighted the significant enrichment of the JAK/STAT signaling pathway (Figure S6I), which has been reported as a downstream effector of the CD44 receptor and induce CXCL1 expression [35, 36]. Consistent with these findings, SPP1 stimulation increased the phosphorylation of STAT3 in MLE-12 cells, and the STAT3 inhibitor Stattic reduced SPP1-induced CXCL1 expression (Fig. 4K, L). Moreover, in vivo experiments also showed that SPP1 administration significantly upregulated STAT3 phosphorylation and CXCL1 expression (Fig. 4M). Similar outcomes were observed in the A549 cell line (Figure S6J-L).

Intriguingly, we noted that SPP1 (encoding SPP1) was among the upregulated genes in the RNA-seq data of both SPP1-stimulated MLE-12 cells and SPP1-PMN (Fig. 4N). The triggered increase in SPP1 level in SPP1-treated MLE-12 cells was verified by qRT-PCR and ELISA (Fig. 4O–P). These findings suggest that tumor-derived SPP1 exerts a local self-amplification through lung epithelial cells, which further increases CXCL1 secretion and neutrophil infiltration in the lung-specific PMN (Fig. 4Q).

### NETs facilitate HCC lung metastasis in SPP1-initiated PMN

**Fig. 5:**
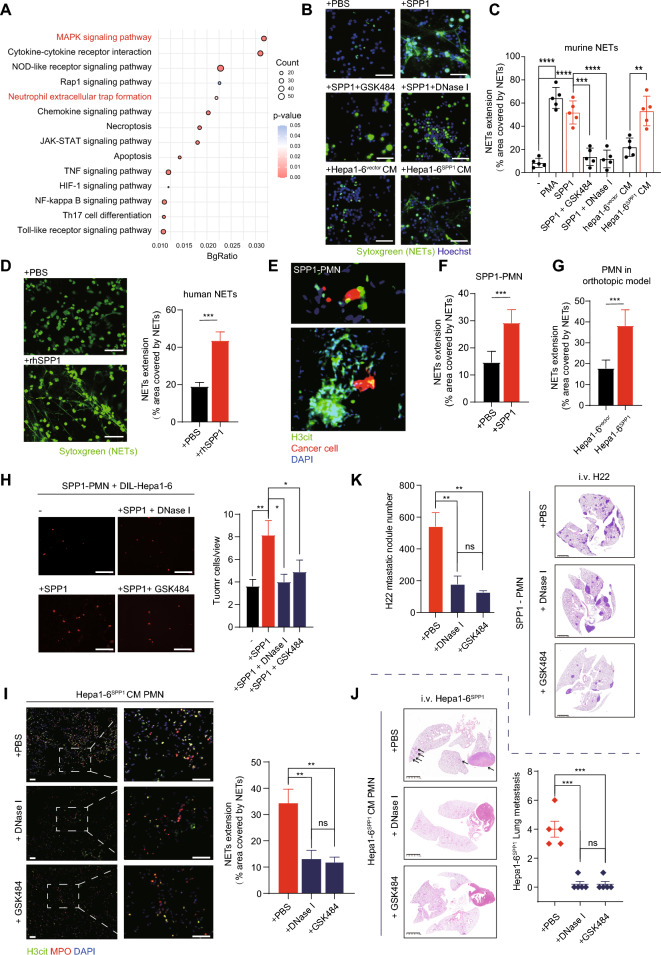
SPP1 induces NETs formation in PMN. **A** KEGG enrichment bubble diagrams of upregulated genes in SPP1-stimulated neutrophils. **B**, **C** Representative IF images of NETs formation (**B**) from mice neutrophils with different administrations. NETs are stained with Sytoxgreen (green), and DNA is stained with Hoechst (blue). To quantify, NETs extension from five random views is shown (**C**). The *P* values were calculated by one-way ANOVA. Scale bar, 100 μm. **D** Representative IF images and quantified NETs percentage from human neutrophils treated with PBS or rhSPP1. The *P* values were calculated by Student’s *t*-test. Scale bar, 100 μm. **E** IF images of DIL-labeled Hepa1-6 cells trapped within NETs. **F**, **G** Quantified NETs extension in lungs of SPP1-PMN at day 7 (F, related to Fig. 1C) and in lungs of Hepa1-6 orthotopic model at day 14 (G, related to Fig. 1G). The *P* values were calculated by Student’s *t*-test. **H** Representative images of adhered DIL-labeled Hepa1-6 cells in SPP1-PMN with the treatment of GSK484 or DNase I, the lungs were harvested 1 day after i.v. injected Hepa1-6 cells. To quantify, tumor cells were counted in five random views for each mouse and took an average count (n = 8 per group). The *P* values were calculated by one-way ANOVA. Scare bar, 100 μm. **I**, **J** The effect of DNase I or GSK484 on experimental metastasis model of Hepa1-6^SPP1^. Representative images of H3cit + and MPO + signals in lung tissues (**I**) and lung metastasis lesions (**J**). The *P* values were calculated by one-way ANOVA. Scale bar, 100 μm. **K** The effect of DNase I or GSK484 on SPP1-PMN model of H22. The lungs were harvested at 14 days after i.v. injected (n = 5 per group). The *P* values were calculated by one-way ANOVA. For all tests, significance was determined with a 95% confidence interval (ns, P > 0.05; *P < 0.05; **P < 0.01; ***P < 0.001; ****P < 0.0001)

To further elucidate the mechanism by which neutrophil-dominant PMN drives HCC lung metastasis, we studied the phenotypic changes of neutrophils in SPP1-orchestrated lungs. RNA-seq analysis of SPP1-stimulated murine neutrophils revealed that these cells could not be classified into classic anti-tumor N1-like or pro-tumor N2-like neutrophils (Figure S7A, Table S5). Instead, significantly upregulated genes enriched in NETs formation were revealed by KEGG pathway enrichment analysis (Fig. 5A). We have previously shown that NETs promote HCC metastasis by enhancing early seeding and promoting a tumorous inflammatory response [26, 37]. We thus speculated that NETs may participate in the SPP1-orchestrated PMN in HCC. In vitro, SPP1 and CM from Hepa1-6^SPP1^ cells led to significant NETs formation in murine neutrophils (Fig. 5B, C), and this effect could be abrogated by the administration of GSK484 (which inhibits NETs formation) or DNase I (which destroys formed NETs) (Fig. 5C) [38, 39]. Consistently, NETs formation in human peripheral blood neutrophils was also increased by rhSPP1 in vitro (Fig. 5D). The ERK and AKT pathways have been reported to be involved in NETs formation, and KEGG analysis of SPP1-stimulated neutrophils also revealed the top enriched MAPK (ERK-involved) signaling pathway (Fig. 5A). Moreover, in vitro inhibition of the ERK pathway, rather than the AKT pathway, significantly reduced NETs formation (Figure S7B-C), indicating that SPP1 facilitated mouse NETs formation by activating the ERK pathway in neutrophils.

For additional in vivo evidence of NETs in SPP1-orchestrated PMN, IF was performed on SPP1-PMN following i.v. injection of DIL-stained Hepa1-6 cells (related to Fig. 1D), which visualized the increased well-like structural NETs trapping Hepa1-6 cells (Fig. 5E, F). In line, this finding was corroborated by increased citrullinated histone H3 (H3cit, a NETs marker) in SPP1-PMN (Figure S7D). In the modified HCC orthotopic model, Hepa1-6^SPP1^ PMN also presented increased NETs formation, as revealed by two-photon imaging and immunoblotting (Fig. 5G, Figure S7E). However, neutralizing SPP1 effectively prevented the increased pulmonary NETs formation in the Hepa1-6^SPP1^-CM treated lungs (Figure S7F). We further asked whether targeting NETs impaired SPP1-induced HCC PMN development and metastasis. As expected, targeting NETs with GSK484 or DNase I efficiently decreased the early seeding of Hepa1-6 cells in SPP1-PMN (Fig. 5H). GSK484 or DNase I efficiently degraded NETs in PMN induced by Hepa1-6^SPP1^ CM and subsequently diminished the experimental lung metastasis of Hepa1-6^SPP1^ cells (Fig. 5I, J). Consistently, H22 metastasis favored by SPP1-PMN was also abrogated by targeting NETs (Fig. 5K). These results indicate the key role of NETs formation in the SPP1-orchestrated HCC PMN.

### Early intervention in SPP1-orchestrated PMN by targeting CXCR2 and NETs prevents HCC lung metastasis

**Fig. 6:**
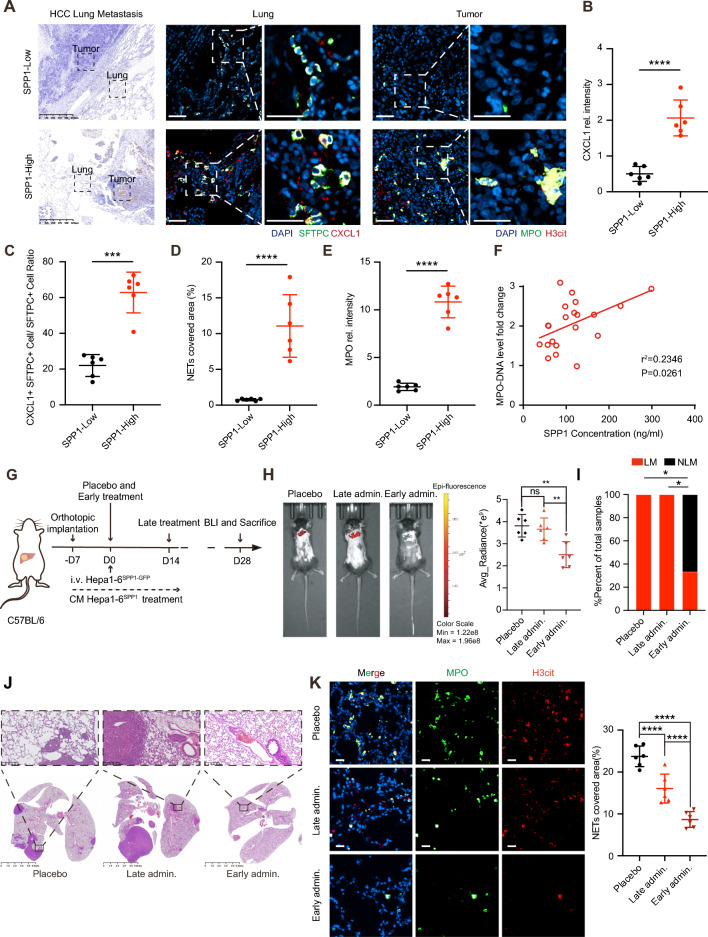
CXCR2 inhibitor plus DNase I treatment inhibits HCC lung metastasis. **A** IHC staining of SPP1 in HCC lung metastasis lesions from Huashan cohort (left). Matched IF staining of CXCL1 + and SFTPC + cells in lung tissues (middle), and IF staining of MPO + and H3cit + cells in metastatic tumor lesions (right). Scare bar, 25 μm. **B**–**E** Quantification analysis of CXCL1 (**B**), percent of CXCL1 + SFTPC + cells (**C**), NETs formation (H3cit^+^, **D**), and Neutrophils (MPO^+^ **E**) in SPP1-high/-low groups (n = 6 per group). The P values were calculated by Student’s *t*-test. Scare bar, 25 μm. **F** Correlation analysis between serum MPO-DNA and SPP1 concentration in HCC samples (n = 21, Pearson correlation). **G** Experimental setup. Briefly, GFP-labeled Hepa1-6 (Hepa1-6^GFP^) cells were i.v. injected into C57BL/6 mice bearing orthotopic Hepa1-6 (unlabeled) at their PMN stage. For the early and late treatment groups, DNase I and CXCR2 inhibitor-SB225002 were given at day 0 and day 14, respectively. At the endpoint, the lung metastatic burden of Hepa1-6^SPP1−GFP^ was detected by BLI (n = 6 per group). **H** Tumor metastasis burden was observed and quantified by fluorescence imaging. Representative lung images (left) and quantified results (right) were shown, respectively. Data were means ± SD. The *P* values were calculated by one-way ANOVA. **I**, **J** Quantification analysis (I) and HE images (J) of lung metastasis lesions. The *P* values were calculated by Fisher’s exact test, one-tailed. **K** The immunostaining of MPO + and H3cit + and the signal intensity were quantified and analyzed, and representative images of indicated staining of lungs were shown. The *P* values were calculated by one-way ANOVA. Scale bar, 50 μm. For all tests, significance was determined with a 95% confidence interval (ns, P > 0.05; **P < 0.01; ****P < 0.0001)

To further validate the roles of the SPP1/CXCL1/NETs axis in HCC lung metastasis, we assessed the clinical relevance of SPP1 and CXCL1 expression in the lung metastasis loci of human HCCs. We found that the metastatic lesions with high SPP1 expression presented elevated CXCL1 levels, and most of them were co-localized with AECIIs (SFTPC^+^) (Fig. 6A–C). Furthermore, we observed increased neutrophil infiltration and NETs formation in the high SPP1 expression group (Fig. 6A, D, E). Meanwhile, the clinical cohort revealed a positive correlation between plasma SPP1 concentration and the circulating NETs level, which was marked by MPO-DNA (Fig. 6F).

Considering the important effects of neutrophils and NETs on HCC lung metastasis, we assessed the therapeutic value and intervention timing of targeting neutrophil recruitment and NETs formation to block SPP1-initiated PMN formation. We established an orthotopic implantation model with Hepa1-6^SPP1^ to generate an SPP1-initiated PMN, followed by i.v loading of small numbers of GFP-labeled Hepa1-6^SPP1^ (Hepa1-6^SPP1−GFP^) cells to mimic the dissemination and metastatic process (Fig. 6G) [40]. Early intervention prior to the establishment of the PMN by combining the inhibition of CXCL1-CXCR2 with SB225002 and destruction of NETs with DNase I effectively diminished pulmonary neutrophil infiltration, NETs formation, and pre-established lung metastasis. In contrast, late intervention after PMN establishment failed to reduce lung metastasis (Fig. 6H–K). Both early and late interventions had no direct effect on orthotopic tumor growth (Figure S8A-B). To further verify the above findings, we repeated the above experiments in human MHCC-97H cells. Orthotopic implantation and i.v injection models were established in nude mice via MHCC-97H cells and luciferase-labeled MHCC-97H cells (MHCC97H^luci^), respectively (Figure S8C). Administration of the CXCR2 inhibitor and DNase I had no effect on primary tumor growth (Figure S8D-E), but only early treatment attenuated the lung metastasis burden (Figure S8F-G).

**Fig. 7:**
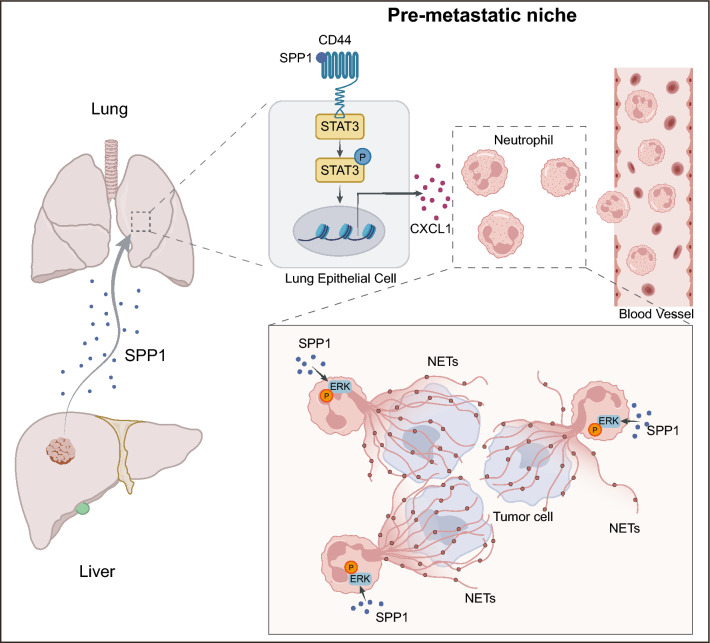
Schematic diagram. In HCC, primary tumor-derived SPP1 stimulated lung epithelial cells to secrete more CXCL1 through SPP1/CD44/STAT3 axis. Elevated CXCL1 levels in the lung microenvironment promoted neutrophil infiltration and NETs formation to facilitate HCC lung metastasis

In conclusion, these data suggest that targeting CXCL1-CXCR2 and NETs formation could effectively prevent lung metastasis in SPP1-positive HCC patients, but early intervention is crucial for successful treatment (Fig. 7).

## Discussion

PMN formation is generally acknowledged as a critical step in cancer metastasis, but how HCC modifies the PMN and facilitates subsequent metastasis remains obscure. Our previous work revealed that SPP1 was a leading pro-metastasis gene in HCC [10], but how HCC-derived SPP1 modified the PMN in target organs, especially in lungs, to fuel the metastasis of liver cancer remains to be further investigated. In this study, we demonstrated that tumor-derived SPP1 could increase lung neutrophil infiltration to form the PMN and subsequently stimulate the formation of NETs to promote HCC lung metastasis. Mechanistically, SPP1 can specifically target lung host cells, known as lung epithelial cells, to secrete CXCL1 through the SPP1/CD44/STAT3 axis and recruit neutrophils to form NETs.

SPP1, a pro-metastatic protein, has been widely studied and is closely related to immune escape, EMT, and other functions required for tumor metastasis [13, 41]. In accordance with these findings, we found that SPP1 was upregulated in tumor tissue compared with matched para-cancerous tissues and was more highly expressed in the tissues and serum of patients with lung metastasis. However, we noticed the plasma SPP1 levels in some patients in the NLM group were higher than the average level of LM group. Previous studies indicated that the SPP1 plasma levels were influenced by Edmondson grading, TNM staging and the number of tumor nodules of HCC patients [11], which made plasma SPP1 levels vary from patient to patient in the same group. Meanwhile, cancer metastasis is a muti-steps and complex process, SPP1 could increase the opportunity of metastasis rather than necessarily lead to HCC lung metastasis. Moreover, tumor-derived SPP1 promoted neutrophil-dominant PMN formation by specifically targeting lung epithelial cells to secrete CXCL1 and subsequently boosted HCC lung metastasis through NETs formation. However, some studies have shown that SPP1 promotes HCC cell proliferation and migration [42], while we found SPP1 overexpression had little effect on Hepa1-6 proliferation and migration (Figure S2A-C). The discrepancy between our study and that of Wu et al. may be due to differences in cell lines, and the addition of SPP1 may not be essential for malignant phenotypes in some HCC cell lines. Meanwhile, we emphasized the role of secretory SPP1 on the PMN rather than the cancer cell itself in this study.

The "soil and seed" theory, a widely recognized concept of cancer metastasis, proposes two critical steps that contribute to the development of metastasis: primary tumors with high metastatic potential and conducive environments in targeted organs [5]. Of particular interest is the formation of PMN, which has garnered significant attention. PMN plays crucial roles in attracting circulating tumor cells, facilitating their survival, reactivating dormant cells, and fueling subsequent outgrowth. In the present study, we revealed that neutrophils significantly accumulated in the lung PMN after SPP1 stimulation and that the neutrophil-depleting Ly6G antibody had a potent ability to suppress lung metastasis. Neutrophil, a pivotal component of the PMN, in primary tumors and target organs is closely related to more metastatic events and worse clinical outcomes in several cancers [21, 43]. And certain neutrophil-recruiting chemokines, such as CXCL1 and CXCL5, were found to be elevated in the serum of patients with HCC [44], also indicating the crucial role of neutrophils in HCC progression. However, the function of neutrophils in tumor progression is controversial [20]. With the advancement of single-cell sequencing technologies, neutrophil heterogeneity in cancers has been revealed, and some studies have validated the dual function of neutrophils in cancer progression in different subsets [45, 46]. Therefore, the conflicting conclusions on neutrophil function in previous articles cannot be attributed entirely to diverse tumor types, and other functions of neutrophil subpopulations should also be further studied.

Previous studies have indicated that exogenous SPP1 induces neutrophil chemotaxis [47]. In our research, we found that SPP1 had a slight chemotactic effect on neutrophils in vitro, and this effect seemed insufficient to cause a significant accumulation of neutrophils in vivo (Fig. 3A). We further found that AECII was the significant cell type that responded to SPP1, and AECII was the main source of SPP1-induced CXCL1 elevation. AECII cells are the most abundant cell type in the lung and are the progenitor cells of type I alveolar epithelial cells, which are closely related to inflammatory diseases [48]. AECII can secrete various chemokines and cytokines to recruit immune cells to phagocytes and kill pathogens or infected/apoptotic cells to maintain lung homeostasis [49]. A previous study revealed that AECII could respond to tumor-derived RNA to recruit neutrophils by secreting chemokines in the PMN [23]. In the present study, AECII increased CXCL1 secretion to recruit more neutrophils from circulation under SPP1 stimulation. Moreover, loss-of-function studies revealed that CD44 was the primary receptor for SPP1 in MLE-12 cells. After injecting his-tag-labeled SPP1, we traced the distribution of SPP1 in different organs and observed that the lung had the highest accumulation abundance. These data showed that the unbalanced distribution of tumor-derived SPP1 may explain the organ-specific metastasis of HCC. Lung epithelial cells highly express the CD44 receptor and activation of the downstream chemotaxis signaling pathway supports PMN formation and promotes subsequent HCC lung metastasis.

Another important question is how recruited neutrophils promote the lung metastasis of HCC. In vitro and in vivo studies revealed that more NETs were formed under SPP1 stimulation, which was associated with a greater lung metastasis burden. The role of NETs in cancer progression is still unclear. While some studies suggest an anti-tumor function of NETs, more studies have shown the protumor function of NETs, especially in supporting tumor metastasis [50]. Our previous study demonstrated that NETs can capture HCC cells and increase their metastatic potential by activating the TLR4/9-COX2 axis [26]. Another study by our team evaluated NETs enrichment levels based on 23 NETs-related genes in 22 cancers and reported that the combination of SPP1 upregulation and NETs enrichment was closely related to EMT in cancers [37]. Moreover, NETs are also involved in many steps of metastasis, for example, Yang et al. showed that CCDC25 on the surface of the tumor cell membrane could sense extracellular DNA and fuel breast cancer cells metastasize to the liver, which contains enriched NETs [22]. The enrichment of NETs in target organs is positively correlated with tumor metastasis, and serum NETs DNA, which is usually measured by MPO-DNA, has also become a potential biomarker for predicting cancer metastasis. In this study, we also found a positive correlation between the MPO-DNA and plasma SPP1 concentration. Elevated plasma SPP1 levels may predict the risk of HCC lung metastasis.

SPP1 has been confirmed as an effective molecule promoting tumor progression and metastasis in various cancers. While SPP1 is indeed a promising therapeutic target, it is currently not feasible to intervene. On the one hand, SPP1 has many important biological functions, including mucosa repair process [51], regulating bone resorption and matrix turnover [52], and activating and regulating various immune cells [53], which makes directly targeting SPP1 can lead to severe side effects and off-target effect. On the other hand, SPP1 is too complex to develop drugs due to transcriptional and post-translational modifications, splice variants, and single nucleotide polymorphisms. The clinical studies related to SPP1 are limited in disease prediction and diagnosis, such as metastatic breast cancer (NCT04274504) and mesothelioma (NCT02029105). However, we think there are possibilities for future exploration. In this study, we revealed exogenous SPP1 could induce endogenous SPP1 expression upregulation (Fig. 4N-Q). The self-amplification mechanism of SPP1 could be a potential therapeutic target and requires further study. In addition, several studies have indicated the presence of structural variations between tumor-derived SPP1 and other SPP1, which suggested a potential avenue for directly targeting tumor-derived SPP1 in the future [54]. Nonetheless, additional research is necessary to substantiate these preliminary observations. In this manner, we elected to intervene at the pivotal stage of tumor metastasis, the PMN.

CXCR2 inhibitors and DNase I have proven safe in clinical practice, and they may be effective interventions that target SPP1-induced PMN formation and arrest HCC lung metastasis. CXCR2 is one of the most important molecules controlling neutrophil activation and migration. CXCR2 antagonists were initially designed to cure pulmonary inflammatory diseases and are gradually being used in cancer treatment with some success in preclinical studies (NCT02499328, NCT02583477). DNase I is a classical destroyer of NETs and extracellular chromatin and has been used in SLE and cystic fibrosis [39]. However, due to its incomplete degradation of NETs, DNase I is usually combined with additional agents. Because pulmonary neutrophil accumulation in the PMN and NETs promoted HCC lung metastasis, we used the CXCR2 antagonists SB225002 and DNase I, which target neutrophil infiltration and NETs formation, respectively, and showed that early intervention could significantly reduce the lung metastasis burden. In contrast, the late intervention failed (Fig. 6G). These data emphasized a therapeutic window beyond which the antimetastatic effects of anti-neutrophil and anti-NETs treatment were attenuated. Given the slight effects of NETs on the growth of HCC, which are supported by the orthotopic implantation results (Figure S8A-B, D-E), we reasoned the efficacy gap is mainly due to the crucial role of neutrophil and NETs in capturing and colonizing circulating tumor cells during the initial phases of metastasis in target organs (Fig. 5E). This notion is supported by some other research, which has shown that neutrophils are more important in the early stage of lung metastasis, whereas macrophages facilitate the proliferation of disseminated cells [55, 56]. And Albrengues J et al. reported that NETs can awaken dormant tumor cells and facilitate metastasis [57]. This study increased our understanding of CXCR2 antagonists and revealed that early administration of CXCR2 antagonists with DNase I may become a potential therapeutic strategy for preventing HCC lung metastasis, especially in SPP1-positive HCC patients.

## Supplementary Information

Supplementary Material 1.

Supplementary Material 2.

Supplementary Material 3.

Supplementary Material 4.

Supplementary Material 5.

Supplementary Material 6.

Supplementary Material 7.

Supplementary Material 8.

## Data Availability

All data generated or analyzed in this study are involved in this published article or its additional files.

## Abbreviations

AEC II: Type II alveolar epithelial cells
CM: Conditioned mediums
CXCL1: C-X-C motif chemokine ligand 1
CXCR2: C-X-C motif chemokine receptor 2
CyTOF: Mass cytometry
FC: Flow cytometry
HCC: Hepatocellular carcinoma
H3cit: Citrullinated Histone H3
IF: Immunofluorescence staining
IHC: Immunohistochemistry
KEGG: Kyoto Encyclopedia of Genes and Genomes
LM: Lung metastasis
MPO: Myeloperoxidase
NETs: Neutrophil extracellular traps
NLM: Non-lung metastasis
OPN: Osteopontin
OS: Overall survival
PMN: Pre-metastatic niche
qRT-PCR: Quantitative real-time polymerase chain reaction
rmSPP1: Recombinant mouse SPP1
RFS: Relapse-free survival
RNA-seq: RNA sequencing
RT: Room temperature
SFTPC: Surfactant Protein C
