# Supplementary material for: Targeting SPP1-orchestrated neutrophil extracellular traps-dominant pre-metastatic niche reduced HCC lung metastasis
Source: Exp Hematol Oncol. 2024 Nov 11;13:111. doi: 10.1186/s40164-024-00571-x (PMC11556024; doi:10.1186/s40164-024-00571-x)
Supplement: Supplementary file 1 — Supplementary Material 1. [file 40164_2024_571_MOESM1_ESM.docx]

**Supplementary Materials**

**Title: Targeting SPP1-orchestrated neutrophil extracellular traps-dominant pre-metastatic niche reduced HCC lung metastasis**

**Running Title:** SPP1 fuels HCC lung metastasis by modifying PMN.

**Authors’ name:** Sun-Zhe Xie^#1,2^, Lu-Yu Yang^#^*^1,2^, Ran Wei^#1,2^, Xiao-Tian Shen^1,2^, Jun-Jie Pan^1,2^, Shi-Zhe Yu^1,2^, Chen Zhang^1,2^, Hao Xu^1,2^, Jian-Feng Xu^1,2^, Xin Zheng^1,2^, Hao Wang^1,2^, Ying-Han Su^1,2^, Hao-Ting Sun^1,2^, Lu Lu^1,2^, Ming Lu^3^, Wen-Wei Zhu*^1,2^, Lun-Xiu Qin*^1,2^

**Affiliations:**

^1^ Hepatobiliary Surgery, Department of General Surgery, Huashan Hospital, Fudan University, Shanghai 200040, China

^2^ Cancer Metastasis Institute, Fudan University, Shanghai 200040, China

^3^ CAS Key Laboratory of Tissue Microenvironment and Tumor, Shanghai Institute of Nutrition and Health, Chinese Academy of Sciences, Shanghai 200031, China

^#^ These authors contributed equally to this manuscript

*** Corresponding authors:**

Lun-Xiu Qin, MD, PhD,

Hepatobiliary Surgery, Department of General Surgery, Huashan Hospital & Cancer Metastasis Institute, Fudan University, 12 Urumqi Road (M), Shanghai 200040, China, E-mail: qinlx@fudan.edu.cn; Tel & Fax: +86-21-52887172.

Wen-Wei Zhu, PhD,

Hepatobiliary Surgery, Department of General Surgery, Huashan Hospital & Cancer Metastasis Institute, Fudan University, 12 Urumqi Road (M), Shanghai 200040, China, E-mail: westoolife@163.com; Tel & Fax: +86-21-52887172.

Lu-Yu Yang, MD

Hepatobiliary Surgery, Department of General Surgery, Huashan Hospital & Cancer Metastasis Institute, Fudan University, 12 Urumqi Road (M), Shanghai 200040, China, E-mail: yangluyu@huashan.org.cn; Tel & Fax: +86-21-52887172.

**Supplementary Methods**

**Mice model: subcutaneous xenograft models**

Subcutaneous xenograft models were established by subcutaneously injecting 2x10^6^ Hepa1-6 or MHCC97H into 6-week-old male mice. Every two days, the tumor's length and width were measured with a vernier caliper and calculated using the formula: volume= (Length x Width^2^)/2. We emphasized that mice were sacrificed, and tumors were dissected when tumor length exceeded 2.0 cm in any dimension.

**Construction of plasmid**

For construction of lentivirus-mediated gene overexpression plasmid, Mouse SPP1 isoform4 (NM_001204233.1) and Mouse SPP1 isoform 2 (NM_001204202.1) cDNA was amplified by PCR and cloned into the downstream of tag in lentivirus vectors pCDH-ZsGreen. Primer sequences were listed as follows: SPP1 isoform 2: F:5’-ccg gaa ttc atg agg ctg cag ttc tcc tg-3’ and R:5’-gcg gat cct tag ttg acc tca gaa gat gaa ctc t-3’; SPP1 isoform 4: F:5’-ccg gaa ttc atg aga ttg gca gtg att tgc t-3’ and R: 5’-gcg gat ccg ttg acc tca gaa gat gaa ctc tct-3’. For the construction of lentivirus-mediated knockdown plasmid, Oligo DNAs containing the short hairpin RNA target sequence (mouse CD44(NM_009851.2), mouse ITGB1 (NM_010578.2), mouse ITGA5 (NM_001314041.1) and mouse ITGAV (NM_008402) -F and -R, **Table S6**) were annealed and cloned into vectors plko.1. The sequences were confirmed by DNA sequencing.

**Lentivirus-mediated knockdown and overexpression of gene expression**

The day before transfection, 5x10^6^ HEK 293T cells were cultured in 10cm dish in DMEM (Gibco, Cat# 11965118, with 10% FBS (Gibco, Cat# 10270106)) for following lentivirus generation. After gently washing three times with 1xPBS (Servicebio, Cat# G4202), HEK 293T cells were transfected with 6 mL serum-free DMEM containing 20 μl Lipofiter (Hanbio, Cat# HB-LF-1000), 10 mg plasmid, which contained target genes or short hairpin structures, combined with 7.5 mg psPAX2 and 2.5 mg pMD2.G at 37 ℃ with 5% CO_2_. After 6 hours, replace medium to 13 mL DMEM with 10% FBS. After 48 hours, supernatant containing lentivirus was collected and centrifuged at 2000 g for 5 min at 4 °C. Then, it was filtered with 0.22 μm filters (Millipore, Cat# SLGPR33RB) and used to infect Hepa1-6 or MLE-12 cell lines. The stable cell lines were obtained by puromycin (InvivoGene, Cat# anti-pr-1) selection for at least 2 weeks.

**Conditioned medium collection**

For conditioned medium (CM) collection, cells were grown to ~80% confluence in 10 cm culture dishes. After three times washing with 1xPBS, cells were cultured in serum-free medium at 37°C for 24 hours. Conditioned media was collected and centrifuged at 2000 g for 10 min, filtrated through 0.22um filter (PALL, Cat# PN4612) to remove debris, and stored at -80°C without repeated freeze.

**Western blot**

Cultured cells were washed with ice-cold 1ml 1XPBS three times, collected by scraper and then lysed with lysis buffer (Beyotime, P0013C, with phosphatase inhibitors (MCE, Cat# HY-K0010) and protease inhibitors (MCE, Cat# HY-K0021, Cat# HY-K0022). The lysate was aspirated about 15 times with a 1 ml syringe, before an additional 10 min of lysis at 4°C, followed by centrifugation at 12000 g for 15 mins. After removing the insoluble material, the protein concentration was measured with BCA protein assay kit (Thermofisher, Cat# 23225). SDS-PAGE loading buffer (Beyotime, Cat# P0015) was added to lysate and boiled for 10 mins. Proteins were separated on 8-15% SDS-polyacrylamide gel (SDS-PAGE) and transferred onto polyvinylidene fluoride (PVDF) membranes (Millipore, Cat# IPVH00010). After blocking with 10% non-fat milk (Beyotime, Cat# P0216) in TBS-T, membranes were incubated with the primary antibody overnight at 4°C with gentle shaking. The primary antibodies for immunoblot were listed in **Table S7**.

**Quantitive RT-PCR**

RNA was extracted using TriZol (Sigma, Cat# T9424) according to the manufacturer’s instructions. 1000 ng RNA was reverse transcribed into cDNA with PrimeScript™ RT Master Mix (Takara, Cat# RR036A). Quantitative RT-PCR was conducted on an ABI PRISM 7900 Sequence Detection System using TB Green Premix Ex Taq™ (Takara, Cat# RR420A) followed by melting curve analysis to verify the specificity of the products. The Ct values of each RNA samples were normalized by subtracting that of control Actin RNA to acquire the ΔCt value and then 2−ΔCt was calculated as the relative mRNA level. Primer sets for quantitative RT-PCR were listed in **Table S6**.

**Recombinant Protein Production**

For in vitro protein expression, the pET-28a-mouse SPP1 plasmid was transformed into BL-21(DE3) E. coli (Beyotime, Cat# D1013S) and cultured in the lysogeny broth (LB) agar plate with 50 μg/ml kanamycin (Beyotime, Cat# ST101) for 18 hours. A single clone was selected and incubated in 5 ml LB liquid (with 50 μg/ml kanamycin) and incubated overnight at 37°C in a shaker at 200 rpm. The incubated E.coli suspension was transferred to 1000 ml LB liquid and incubated at 37°C for 4~6 hours and then 0.5 mM isopropyl-β-D-thiogalactopyranoside (IPTG, MCE, Cat# HY-15921) was added at 20°C for 12 hours to induce the protein expression. E.coli cells were centrifuged at 4000 g for 20 min at 4 °C and then sonicated (300w, ultrasonic 10 s, interval 10 s, total time 10 min) in ice bath. After centrifuging at 10,000 × g for 10 min at 4 °C, the supernatants were collected and using Ni-NTA column (Qiagen, Cat#30210) to purify the recombinant SPP1 protein according to the product instruction. Briefly, After wash steps, recombinant SPP1 protein was eluted by 200 mM and 500 mM imidazole (Sigma, Cat#56705). The purified protein was stored at -80°C.

**Flow cytometry analysis (FC)**

The lungs and tumor tissues were picked immediately when mice were sacrificed. In addition, the lungs were perfused with 20 ml 1XPBS through the right ventricle. Tissues were minced into small pieces and further digested in hanks' balanced salt solution (Beyotime, Cat# C0219) containing 1 mg/ml Collagenase Type I (Sigma, Cat# C1-BIOC), Collagenase Type II (Sigma, Cat# C2-BIOC), Collagenase Type IV (Sigma, Cat# C4-BIOC), and 0.1 mg/ml DNase I (Solarbio, Cat# D8071) at 37°C, 800 rpm shaking for 1 hour. The supernatant was filtered through MACS® SmartStrainers (70 µm, Miltenyibiotec, Cat# 130-110-916). Then, Red blood cell lysis solution (Miltenyibiotec, Cat# 130-094-183) was used to lyse red blood cells. The supernatant was filtered through MACS® SmartStrainers (30 µm, Miltenyibiotec, Cat# 130-098-458) to obtain a single-cell suspension. After incubating for 1 hour at 4°C with 5% BSA, cells were incubated for 30 min at 4°C with FC antibodies, and the antibodies were listed in **Table S1**. FC was performed by Gallious (Beckman) FC system and analyzed using FlowJo V10 software. As for FACS, cells were sorted using MoFlo XDP system (Beckman).

**Two-photon imaging**

In experimental HCC lung metastasis models, SytoxGreen (1:10000, Invitrogen, Cat# S7020) was i.p. injected. After 20 min, mice were sacrificed, and lungs were immediately resected and washed in 1xPBS. Two-photon imaging was conducted using a Zeiss LSM710 (Carl Zeiss) coupled with a W Plan-Apochromat 20×/1.0 DIC water immersion objective (Carl Zeiss) and a Chameleon femtosecond laser. The excitation wavelength was set to 880 nm. SytoxGreen photons were collected using two emission filters and a beam splitter. For visualizing collagen structures, signals for second-harmonic generation were recorded between 410-455 nm.

**Gene expression analysis of the TCGA database**

RNA-sequencing expression (level 3) profiles and corresponding clinical information were downloaded from the TCGA dataset. The current-release (V8) GTEx datasets were obtained from the GTEx data portal website (<https://www.gtexportal.org/home/datasets>). Statistical analyses were performed using R software v4.0.3 (R Foundation for Statistical Computing, Vienna, Austria). P-value <0.05 was considered statistically significant.

**Supplementary figures and figure legends**

**Figure S1. High expression of SPP1 is correlated with poor prognosis**

(A) mRNA expression of SPP1 was compared between HCC tissues and normal liver tissues from the TCGA-HCC database. The *P* values were calculated by the Wilcox test. (B) Kaplan-Meier survival analysis of low SPP1 (in blue) and high SPP1 (in red) HCC patients in TCGA dataset. “TPM” stands for transcripts per million (TPM), a normalization method employed in bulk RNA sequencing (RNA-seq) to quantify gene expression levels. The *P* value was calculated by the Log-Rank test. (C) qRT-PCR showed the SPP1 levels in samples of human HCC tissues and matched para-tumor tissues. The *P* values were calculated by the student’s *t*-test. (D-E) Representative images of SPP1 RNA scores (D) and SPP1 mRNA expression (E) in HCC tissues. The scores were calculated by the intensity and percentage of stained cells using RNAscope In Situ Hybridization. Scale bar, 100μm. (F) Quantification of SPP1 expression levels according to RNAScope scores in 90 samples of HCC and para-tumor tissues. (G) According to the scores of RNAscope, patients were divided into SPP1-low (score of 0-2, n = 54) and SPP1-High (score of 3-4, n = 36). Shown were Kaplan-Meier survival analysis of the overall survival (OS, left) and recurrence-free survival (RFS, right) of HCC patients based on the two groups. The *P* value was calculated by the Log-Rank test. (H) Linear regression analysis showed a significant association between plasma SPP1 concentration and SPP1 expression levels in HCC tissues (n = 15). (I) SDS-PAGE image of purified recombinant mouse SPP1 (rmSPP1). (J) RNA-seq analysis of PMN-related genes in lung tissues of rmSPP1-PMN models (relater to Fig. 1C). (K-M) The pro-metastatic gene expression in the liver (K), brain(L), colon(M) of mice at 7 days after SPP1 treatment (10mg/kg, i.p.) (n = 6 per group). The *P* values were calculated by Student’s *t*-test. (N-O) Immunoblot (N) and qRT-PCR (O) validated the SPP1 expression in Hepa1-6^vetor^ and Hepa1-6^SPP1^. The *P* value was calculated by the student’s *t*-test. (P) Subcutaneous tumors (right panel) and orthotopic tumors (left panel) of Hepa1-6^vetor^ and Hepa1-6^SPP1^ (related to Fig. 1G). The *P* values were calculated by the student’s *t*-test. For all tests, significance is determined with a 95% confidence interval (ns, P > 0.05; *, P < 0.05; **, P < 0.01; ***, P < 0.001; ****, P < 0.0001).

**Figure S2. Secreted SPP1 and intracellular SPP1 have different effects on HCC in vitro and in vivo.**

**(**A-E) Elisa (A) and qRT-PCR (B) analysis of SPP1 levels of Hepa1-6^vector/SPP1/SPP1△1-48^. The *in vitro* effects of SPP1 and SPP1 (△1-48) on migration (C-D) and proliferation (E) of Hepa1-6 cells. To quantify, the number of migrated cells was counted in five random fields of vision per sample. The *P* values were calculated by two-tailed one-way ANOVA. Scale bar, 100 μm. (F-G) Representative images (F) and quantification (G) of subcutaneous (n = 6 per group), orthotopic (n = 3 per group), lung metastatic loci (n = 6 per group) of Hepa1-6^vector/SPP1/ SPP1△1-48^. The *P* values were calculated by the two-tailed one-way ANOVA (for the subcutaneous and orthotopic model) and the Fisher exact test (for the lung metastasis model). (H) The effect of the anti-SPP1 neutralizing antibody on HCC metastasis was evaluated in the experimental metastasis model of MHCC97H cells through i.v. injection. For all tests, significance was determined with a 95% confidence interval (ns, P > 0.05; *, P < 0.05; **, P < 0.01; ***, P < 0.001; ****, P < 0.0001).

**Figure S3. Characterization of Immune cells in rmSPP1-PMN**

(A) Heatmap showing the median expression levels of markers among the obtained clusters. (B) TSNE plot projection of the expression levels of the CD45, CD3, CD4, CD8a, CD11b, Ly6G, CD19, MHCII, F4/80, Ki67 markers in the obtained clusters. (C) Representative FC gating strategy for quantifying the numbers of various immune cells. (D) Flow cytometry analysis of immune cells in the lung tissues from PBS- or rmSPP1-treated mice (n = 6 per group). The *P* values were calculated by the *t*-test. For all tests, significance was determined with a 95% confidence interval (ns, P > 0.05; *, P < 0.05).

**Figure S4. Tumor-derived SPP1 promotes neutrophil infiltration in the pre-metastatic niche.**

(A) The ImmuCC algorithm analysis on RNA-seq data of rmSPP1-PMN (related to Fig. S1K) and relative fold change compared to the PBS group was shown. (B) Images and quantification of lung metastasis loci of MHCC97H in experimental metastasis model after depleting neutrophils by anti-Ly6G antibody. The *P* values were calculated by the *t*-test. For all tests, significance was determined with a 95% confidence interval (ns, P > 0.05; ***, P < 0.001).

**Figure S5. Lung epithelial cells are the predominant participant cells in SPP1-induced pro-metastasis PMN.**

(A) FC analysis of infiltrating immune cells of peripheral blood in the orthotopic model of Hepa1-6^vector/SPP1^ (n = 4 per group). The *P* values were calculated by the *t*-test. (B) qRT‒PCR analysis of neutrophil-related chemokines and cytokines in lung tissue after i.v. injection of rmSPP1. The *P* values were calculated by the *t*-test. (C) Representative IF images of rmSPP1-his and B cells (CD19^+^), fibroblasts (PDGFRA^+^), neutrophils (Ly6G^+^), T cells (CD3^+^), and macrophages (F4/80^+^) co-location in the lung of mice with i.p administration of rmSPP1-his. Scale bar, 20μm. (D) qRT-PCR analysis of *Cxcl1, Cxcl2,* and *Cxcl3* in freshly purified AEC II cells treated with rmSPP1 or PBS. The *P* values were calculated by the *t*-test. (E) qRT-PCR analysis of several chemokines in A549 cells treated with PBS or rhSPP1. The *P* values were calculated by the one-way ANOVA. For all tests, significance was determined with a 95% confidence interval (ns, P > 0.05; *, P < 0.05; **, P < 0.01; ****, P < 0.0001).

**Figure S6. SPP1 upregulates CXCL1 expression through CD44 receptors in both murine and human lung epithelial cells.**

(A-B) qRT-PCR analysis of SPP1 receptor (*Cd44* and integrin family) expression in whole lung tissue of C57BL/6 (A) and MLE-12 cell line (B). (C-F) Immunoblot (upper panel) and matched grayscale analyses (lower panel) showed the knockdown of CD44 (C), ITGAV (D), ITGB1 (E), an ITGA5 (F) in MLE-12 cells. The grayscale values reflecting the CD44 (C), ITGAV (D), ITGB1(E), ITGA5 (F) levels in the control group were set as 1.0. ImageJ was used in gray value analyses. The *P* values were calculated by the *t*-test. (G) Immunoblot showed the CXCL1 and CD44 levels in MLE-12 cells under different concentrations of rmSPP1 stimulation. (H) Gray scale analyses of bands from experiments as described in G. The grayscale values reflecting the CD44 and CXCL1 levels in the control group were set as 1.0, separately. The *P* values were calculated by the one-way ANOVA. (I) KEGG enrichment bubble diagrams of upregulated genes in rmSPP1-stimulated MLE-12 cells. (J-L) Immunoblot (J), grayscale analyses (K) and qRT-PCR analysis (L) of CXCL1 in CD44 siRNA transferred A549 cells treated with rhSPP1. The *P* values were calculated by the *t*-test. For all tests, significance was determined with a 95% confidence interval (ns, P > 0.05; *, P < 0.05, ***, P < 0.001).

**Figure S7. SPP1 directly stimulates NETs formation of infiltrated neutrophils trough activating Erk pathway.**

(A) Heatmap of N1-like and N2-like genes in murine neutrophils treated with PBS or rmSPP1. (B-C) representative IF images (B) and quantitative analysis (C) of NETs in murine neutrophils treated with ERK- or AKT-inhibitor and rmSPP1 stimulation. The *P* values were calculated by the one-way ANOVA. Scale bars, 50 μm. (D) Representative images of DAPI, MPO, and H3cit staining in lung tissues from mice after i.p injection of rmSPP1 or PBS. Scale bar, 100 μm. (E) Representative two-photon microscopy images and quantitation of NETs formation in the mouse lung tissues after i.v. injection of 1*10^6^ Hepa1-6^vector/SPP1^. The *P* values were calculated by the *t*-test. Scale bar, 60μm. (F) Representative images of DAPI, MPO, and H3cit staining in lung tissues from mice after i.p. injected Hepa1-6^SPP1^-CM plus IgG or anti-SPP1 treatment. Scale bar, 100 μm. For all tests, significance was determined with a 95% confidence interval (ns, P > 0.05; *, P < 0.05; **, P < 0.01; ***, P < 0.001; ****, P < 0.0001).

**Figure S8. Early administration of CXCR2 inhibitor and DNase I inhibits HCC lung metastasis without significant effects on primary tumor growth.**

(A-B) Shown are representative images (A) and quantitative analysis (B) of primary tumor in the experimental lung metastasis model of Hepa1-6. (C) Experimental setup. BALB/C nude mice are orthotopically implanted with MHCC97H beginning 7 days before i.v injection of 5*10^5^ MHCC97H cells. DNase I and CXCR2 inhibitors are given at day 0 and day 14, separately. At the endpoint, the metastatic burden is measured. (D-G) Shown are representative images (D) and quantitative analysis (E) of the primary tumor, representative images (F), and quantitative analysis (G) of metastatic number in the experimental lung metastasis model of MHCC97H. For all tests, all The *P* values were calculated by the one-way ANOVA, and significance was determined with a 95% confidence interval (ns, P > 0.05; *, P < 0.05; **, P < 0.01; ***, P < 0.001; ****, P < 0.0001).
